# Population Pharmacokinetic-pharmacodynamic Model Analysis of Dapagliflozin for HbA1c-lowering Effects in Japanese Patients with Type 2 Diabetes Mellitus using Long-term Real-world Data

**DOI:** 10.7150/ijms.111519

**Published:** 2025-04-22

**Authors:** Shinji Kobuchi, Shuhei Sakai, Ryosuke Terada, Ken-Ichiro Kato, Tetsuo Hayakawa, Toshiyuki Sakaeda

**Affiliations:** 1 Department Pharmacokinetics, Kyoto Pharmaceutical University, Kyoto 607-8414, Japan.; 2 Department of Diabetes Mellitus and Endocrinology, Tonami General Hospital, Toyama 939-1395, Japan.

**Keywords:** real-world, long-term, population PK model analysis, population PK-PD model analysis

## Abstract

**Objectives:** Dapagliflozin, a sodium-glucose cotransporter 2 inhibitor, has demonstrated population-level benefits in patients with various metabolic, cardiovascular, and renal comorbidities. However, significant inter-individual differences exist in plasma exposure and response to dapagliflozin. This study aimed to identify factors influencing the HbA1c-lowering effects of dapagliflozin using long-term real-world data and a population pharmacokinetic-pharmacodynamic (PK-PD) modeling approach.

**Methods:** A PK-PD model was applied to analyze 415 plasma dapagliflozin concentrations and 508 HbA1c measurements from 85 patients with type 2 diabetes mellitus (T2DM) treated with dapagliflozin for one year. The long-term real-world data enabled the evaluation of treatment variability over time. Inter-individual variability in PK-PD parameters was assessed, and covariate analysis was performed to identify patient-specific factors affecting drug response.

**Results:** HbA1c time profiles were well described using the PK-PD turnover model with an E_max_ function. Body weight significantly influenced the apparent clearance of dapagliflozin, though its clinical impact on systemic exposure was minimal. Long-term real-world data analysis revealed substantial inter-individual variability in HbA1c response.

**Conclusion:** By integrating pharmacometric modeling with long-term real-world data, this study provided unique insights into the determinants of dapagliflozin efficacy in routine clinical practice. These findings highlight factors that may not be captured in short-term clinical trials. These findings emphasize the importance of individualized treatment strategies and suggest that future research should incorporate additional covariates, such as variations in glycemic response dynamics, to further refine dose optimization and personalized diabetes management.

## Introduction

Dapagliflozin is a sodium-glucose cotransporter 2 (SGLT2) inhibitor commonly prescribed for the improvement of glycemic control as an adjunctive therapy to diet and exercise in patients with type 2 diabetes mellitus (T2DM). Although it was first approved for T2DM, dapagliflozin has been demonstrated to be beneficial in the treatment of cardiac and renal disease, regardless of the presence or absence of diabetes 1-3. In Japan, the approved dose of dapagliflozin is once-daily 5 mg/day for patients with T2DM, but a once-daily dose of 10 mg/day is needed for patients with chronic heart failure or chronic kidney disease.

Although dapagliflozin shows benefits in various co-morbid metabolic, cardiovascular, and renal conditions at the population level, substantial inter-individual variability exists in dapagliflozin pharmacokinetics and glycemic response, posing challenges for optimizing therapy in real-world clinical practice 4. To manage disease conditions in each patient, it is critical to identify the factors that define the efficacy of dapagliflozin, especially in real-world settings. In our previous study, the long-term (1-year) stability of plasma dapagliflozin concentration (average trough levels of 2-5 ng/mL with intra-patient variability <30%) was found to be important factor for lowering the glycated hemoglobin (HbA1c) level in a once-daily 5 mg/day dapagliflozin treatment to Japanese patients with T2DM 5. However, multivariate analysis did not establish a direct correlation between average plasma concentrations and HbA1c-lowering effects, underscoring the complexity of exposure-response relationships 5. A more comprehensive pharmacometric analysis integrating time profiles of drug exposure and glycemic response is necessary to elucidate the underlying factors influencing therapeutic outcomes.

To better understand the key factors influencing HbA1c-lowering effects and inter-individual variability in glycemic response, the pharmacometrics approaches using population pharmacokinetic (PK)-pharmacodynamic (PD) model provide a robust framework for integrating drug exposure and response over time 6. By accounting for individual differences and time-dependent changes, these models enable a more comprehensive assessment of treatment variability in real-world settings. While previous PK-PD models for other SGLT2 inhibitors, such as canagliflozin and empagliflozin, have been developed from controlled clinical trial data 7-10, they often exclude diverse real-world patient characteristics and may not fully capture long-term treatment effects.

In this study, we aimed to identify factors associated with the HbA1c-lowering effects of dapagliflozin and inter-individual variability in glycemic response using long-term real-world data. A population PK-PD modeling approach was utilized as a tool to quantify drug exposure-response relationships and assess the impact of patient-specific factors. By leveraging real-world data, this study provides clinically relevant insights into optimizing dapagliflozin therapy in a broader patient population, supporting the advancement of personalized diabetes management.

## Materials and methods

### Ethics

The Ethics Committees of Tonami General Hospital (no. 26136) and Kyoto Pharmaceutical University (no. 16-07) reviewed all relevant study documents and approved the study. The study was conducted at the Tonami General Hospital (Toyama, Japan) and Kyoto Pharmaceutical University (Kyoto, Japan). All participants provided written informed consent prior to enrollment. This study was conducted according to the guidelines of the Declaration of Helsinki.

### Data sources

Plasma concentrations of dapagliflozin and clinical laboratory tests (including HbA1c levels) and patient characteristics were obtained from our previous report on 72 Japanese outpatients 5, with 13 additional Japanese outpatients. All patients were diagnosed with T2DM at an early stage of diabetic nephropathy (urinary albumin-to-creatinine ratio < 30 mg/g Cr) or with inadequate glycemic control and were treated with a once-daily oral dose of 5 mg dapagliflozin after breakfast. In Japan, the dose can be increased to a once-daily dose of 10 mg/day if insufficient. However, none of the patients were prescribed this dose. Prior to the initiation of dapagliflozin treatment, all patients received metformin (1000 mg/day or more) and a dipeptidyl peptidase-4 inhibitor daily. Patient visits were scheduled to conduct clinical laboratory tests, vital sign monitoring, and other routine medical inquiries at 1, 3, 6, 9, and 12 months after dapagliflozin prescription. At each visit, patients were instructed to fast overnight. The plasma concentration of dapagliflozin was determined by liquid chromatography-tandem mass spectrometry (LC-MS/MS) coupled with an API 3200 triple quadrupole mass spectrometer (SCIEX, Framingham, Massachusetts, USA). The details were in our previous study 5. The precision for the analytes was < 15% and accuracy was within ± 15%. The lower limit of quantification for the analytes was 0.5 ng/mL in 100 μL of plasma.

### Population PK-PD modeling

The population PK and PK-PD model analyses employed a nonlinear mixed-effects modeling approach using Phoenix NLME software v.8.4 (Certara USA, Inc., Princeton, NJ, USA) for modeling and parameter estimation, with a first-order conditional estimation conducted using the extended least squares method. According to a previous report 8, a population PK-PD model was developed by linking the time profiles of plasma dapagliflozin concentration to the time profiles of HbA1c levels. The ultimate model selection criteria included Akaike's information criterion (AIC), -2 × log likelihood (-2LL), coefficient of variation (CV) of parameter estimates, goodness-of-fit plots, and visual predictive check plots (*n* = 1000). Inter-individual variability in PK and PD parameters was presumed to follow a logarithmic normal distribution with a mean of 0 and a variance of ω^2^ (equation 1):


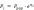

(1)

In equation 1, P*_i_* is the individual parameter estimate for individual *i*, P_pop_ is the population estimate for parameter, and η*_i_* is the inter-individual variability of the *i*th individual. Additive, proportional, and combined (additive and proportional) error models were used to describe residual variability in the observed PK and PD data.

### PK model

Since all plasma dapagliflozin concentrations were measured at trough time points, the absorption rate constant (k_a_) and apparent volume of distribution (V/F) could not be reliably estimated. Therefore, a one-compartment first-order absorption model was applied for the time profiles of plasma dapagliflozin concentrations, and k_a_ and V/F were fixed based on the previous report of population PK analysis 11. The initial values for the apparent clearance (CL/F) in the model parameter estimations were based on previous reports of PK analysis 12 or population PK analysis 11, 13, 14. The estimates included the population mean of CL/F and its inter-individual variability. To evaluate the impacts of covariates on the CL/F of dapagliflozin, a stepwise approach was employed: forward addition (p < 0.01) and backward elimination (p < 0.001) were conducted based on changes in -2LL, and AIC values were also used to assess model adequacy and parsimony. The covariates examined were demographic data (sex, age, height, and body weight), hepatic function (aspartate aminotransferase (AST), alanine transaminase (ALT), and γ-glutamyltransferase (γ-GTP)), and renal function (estimated glomerular filtration rate (eGFR)). The eGFR value was calculated using the serum creatinine level, age, and correction factor for female 15. Continuous covariates were normalized by the mean value of the subjects, and the power or linear model was initially tested.

### PD model

Turnover with the E_max_ model established by de Winter *et al.* 8 was applied to the time profiles of HbA1c levels using the following equations 2 to 5:


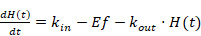

(2)


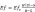

(3)


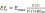

(4)


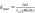

(5)

Equation 2 describes the turnover of HbA1c, where H(t) is the HbA1c level at time t; k_in_ and k_out_ are a zero-order rate constant for HbA1c production through hemoglobin glycation and a first-order rate constant for HbA1c elimination through erythrocyte cell turnover, respectively 16. The Ef is derived from equation 3, and Efc in equation 3 is defined by equation 4. The E_max_ is maximum HbA1c-lowering effect of dapagliflozin for a typical patient with HbA1c at baseline of 8.0%, and the EC_50_ is dapagliflozin plasma exposure (C(t)) at which the half-maximal effect is reached. In equation 3, the HbA1c level was corrected for a lower boundary of 5.0% because normoglycemia is typically associated with that value. The baseline HbA1c value for each patient was set to the corresponding observed value. The estimates included the population mean half-life of HbA1c (t_1/2_HbA1c), E_max_, and EC_50_, after fixing the individual post hoc PK parameters obtained by the PK model analysis. At baseline, dH(0)=0 was assumed and k_in_ was estimated as k_in_ = H(0)/k_out_. We employed an inter-individual variability model for t_1/2_HbA1c, adhering to the specified model development criteria.

### Model evaluation

The final population PK and PK-PD models were evaluated using a prediction-corrected visual predictive check (pc-VPC) and nonparametric bootstrap analysis. For the pc-VPC, the 5th, 50th, and 95th percentiles of plasma dapagliflozin concentrations were simulated to obtain datasets (n = 1000) using the final model parameters. A nonparametric bootstrap procedure (n = 1000) was conducted to compare the parameters with the final model parameters estimated from the original dataset and obtain confidence intervals for the model parameters.

### Simulations of time-profiles of HbA1c level at a once-daily dose of 10 mg/kg

In Japan, the approved dose of dapagliflozin is 5 mg/day; if insufficient, it can be increased to 10 mg/day. However, clinical data on the long-term glycemic effects of 10 mg dapagliflozin in Japanese patients remain limited. To explore the potential impact of this higher dose, we performed simulations of HbA1c time profiles over a one-year treatment period using the established population PK-PD model. The simulated population was generated based on the demographic and clinical characteristics of the real-world dataset used for model development. Inter-individual variability and residual variability from the final model were incorporated into the simulations to reflect real-world variations in drug response. The simulated HbA1c profiles for the 10 mg dose were compared to the model-predicted HbA1c trajectories for the 5 mg dose to evaluate the expected additional glycemic benefit of dose escalation.

### Statistical analysis

Continuous data are presented as mean ± standard deviation (SD) and median with range to comprehensively describe the distribution characteristics of the data. The data at baseline and after 12 months of treatment were compared using Student's paired t-test. Data analyses were performed using Bell Curve in Excel software (Social Survey Research Information Co., Ltd. Tokyo, Japan).

## Results

### Study population

**Figure 1:**
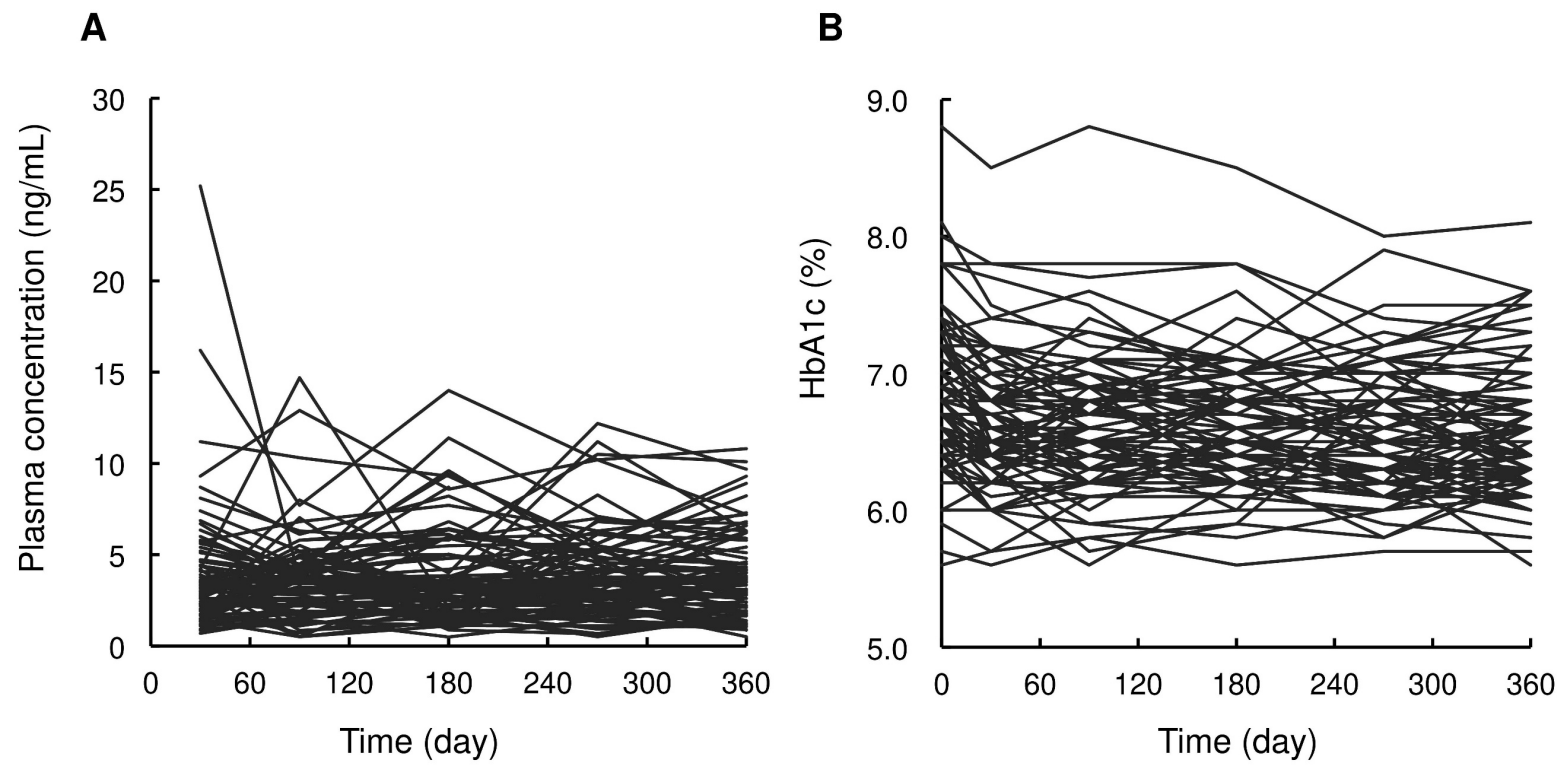
Time profiles of plasma dapagliflozin concentrations (A) and HbA1c levels (B) after oral administration of 5 mg/day of dapagliflozin during the 12 months of treatment.

Patient characteristics are summarized in Table 1. Dapagliflozin was administered to 85 patients. At baseline, the median patient age was 59 years (range: 37 to 75 years), body weight was 77.0 kg (49.0 to 118.0 kg), HbA1c level was 6.8% (5.6 to 8.8%), and eGFR was 74.3 mL/min/1.73 m^2^ (39.2 to 137.8 mL/min/1.73 m^2^). Figure 1 shows the time profiles of plasma dapagliflozin and HbA1c levels. Although extensive inter-individual variability was confirmed for both, HbA1c levels significantly decreased to 6.4% (5.6 to 8.1%) after 12 months of treatment with a once-daily oral dose of 5 mg dapagliflozin. Body weight was also significantly decreased to 74.3 kg (43.0 to 114.0 kg).

### Population PK model

**Figure 2:**
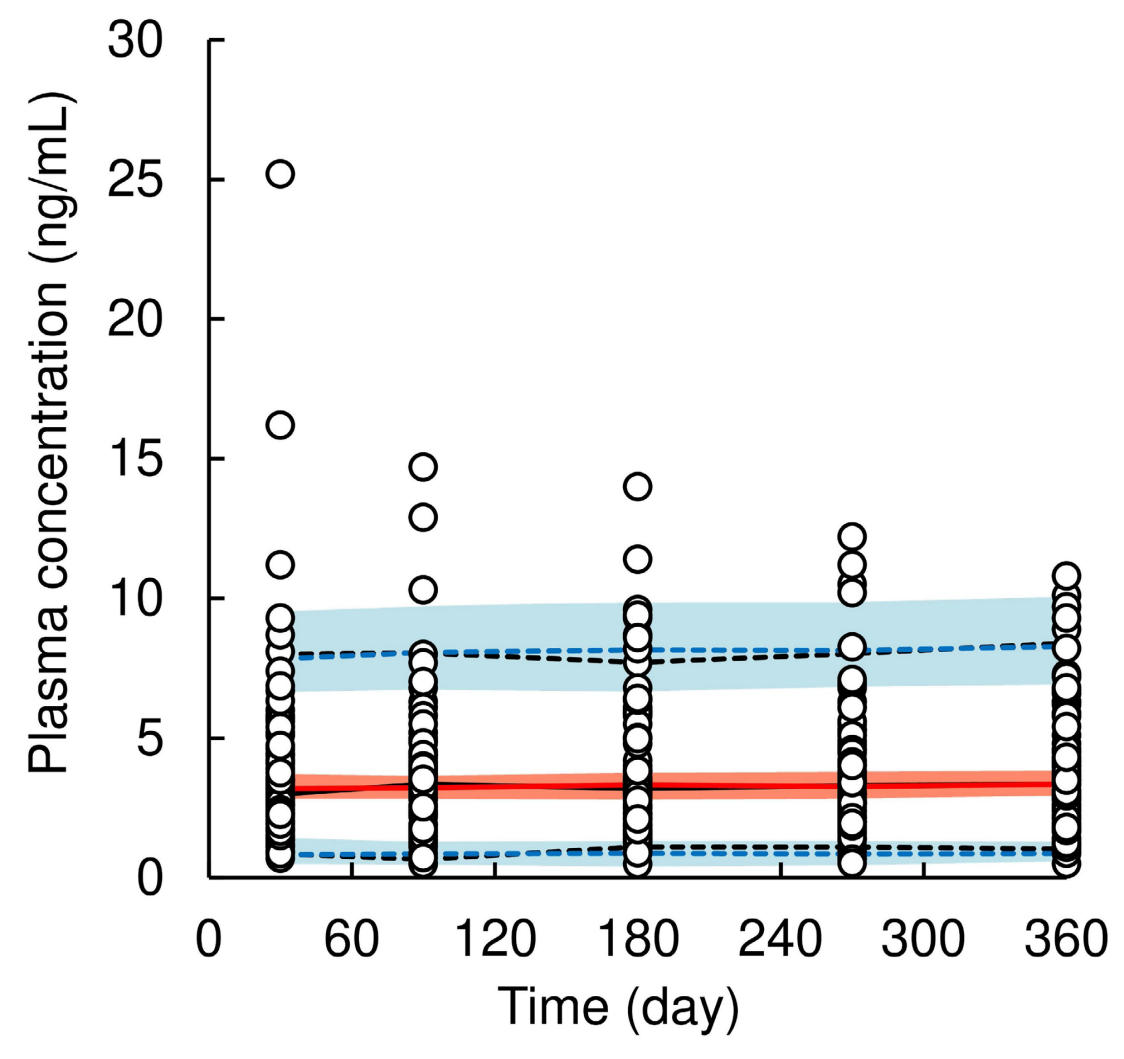
Visual predictive check plots of population pharmacokinetic model of dapagliflozin. Open circles represent observed data of dapagliflozin concentration in plasma. The solid black and red line represents the median observed and simulated plasma concentrations, respectively, and the semitransparent red field represents a simulation-based 95% confidence interval for the median. The observed and simulated 5% and 95% percentiles are presented with dashed black and blue lines, respectively, and the 95% confidence intervals for the corresponding model predicted percentiles are shown as semitransparent blue fields.

A total of 415 plasma concentrations of dapagliflozin from 85 patients were used for population PK model analysis, excluding data under the lower limit of quantification. The pc-VPC plots of the population PK model are presented in Figure 2, goodness-of-fit plots are shown in Supplementary Figure S1, and final population PK parameter estimates are shown in Table 2. A one-compartment model with first-order absorption using multiplicative error correction best described the time profiles of plasma dapagliflozin concentrations. The process of covariate selection from the base model to the final model is summarized in Supplementary Table 1. Covariate analysis identified baseline body weight on CL/F. Because dapagliflozin generally has a weight-lowering effect, we reanalyzed the data by including body weight not only at baseline but also at blood sampling time as a potential covariate and found that body weight at blood sampling time was a significant covariate for CL/F in the final population PK model. The estimated population mean CL/F was 229.3 L/day (1.5% CV), and the final model for CL/F was defined as follows:


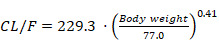

(6)

where 0.41 (2.5%-97.5%CI: 0.24-0.58) is the exponential coefficient of body weight. The CV% of each PK parameter value was ≤ 21.3%, indicating well-estimated parameter. The median values of the population PK parameter estimates obtained using the bootstrap procedure were similar to those obtained from the original dataset. Moreover, for validation of the final model using pc-VPC plots, the simulated predictions were nearly identical to those of the observed data, demonstrating a good fit to the model.

### Population PK-PD model

**Figure 3:**
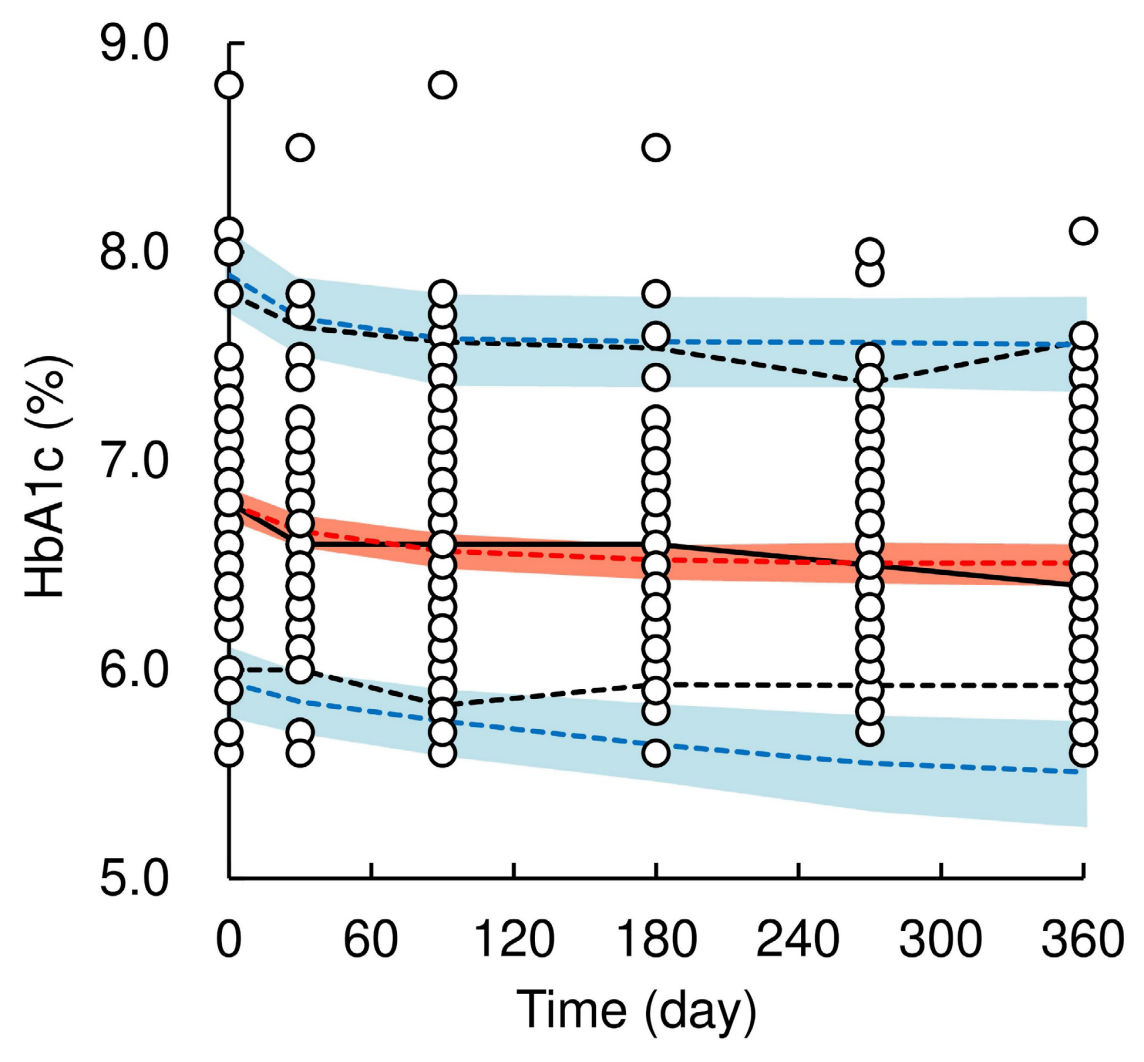
Visual predictive check plots of population pharmacokinetic-pharmacodynamic model of dapagliflozin. Open circles represent observed data of HbA1c level. The solid black and red line represents the median observed and simulated HbA1c levels, respectively, and the semitransparent red field represents a simulation-based 95% confidence interval for the median. The observed and simulated 5% and 95% percentiles are presented with dashed black and blue lines, respectively, and the 95% confidence intervals for the corresponding model predicted percentiles are shown as semitransparent blue fields.

A total of 508 HbA1c values from 85 patients (data at baseline were included) were used for the population PK-PD model analysis. The pc-VPC plots of the population PK-PD model are shown in Figure 3, and the final population PK-PD parameter estimates are summarized in Table 3. The time profiles of the HbA1c levels could be adequately described using a previously reported turnover with the E_max_ model 8 with an additive error correction. The application of the Hill factor to the E_max_ model did not improve the model fit. The final model provided a satisfactory fit to the observed HbA1c data and estimating reliable parameters (CV% ≤ 11.2%). The inter-individual variability of t_1/2_HbA1c was estimated to be 103.9% (CV% = 11.2), which was relatively high. The median value of each model parameter, estimated using the bootstrap procedure, was similar to that of the original dataset. The results of the pc-VPC of the population PK-PD model showed that the observed values largely fell within the median, 5^th^, and 95^th^ percentiles of the simulated data. However, in the later phase after the initiation of dosing (approximately 150 days onward), the lower 5^th^ percentile of the observed values slightly deviated from the predicted 5^th^ percentile confidence interval, indicating a slight overestimation of dapagliflozin's HbA1c-lowering effect. These validation results indicate that the population PK-PD model is generally robust and applicable for simulating HbA1c levels after long-term dapagliflozin treatment in real-world clinical practice; therefore, the final model was retained for model-based simulations.

### Simulation

**Figure 4:**
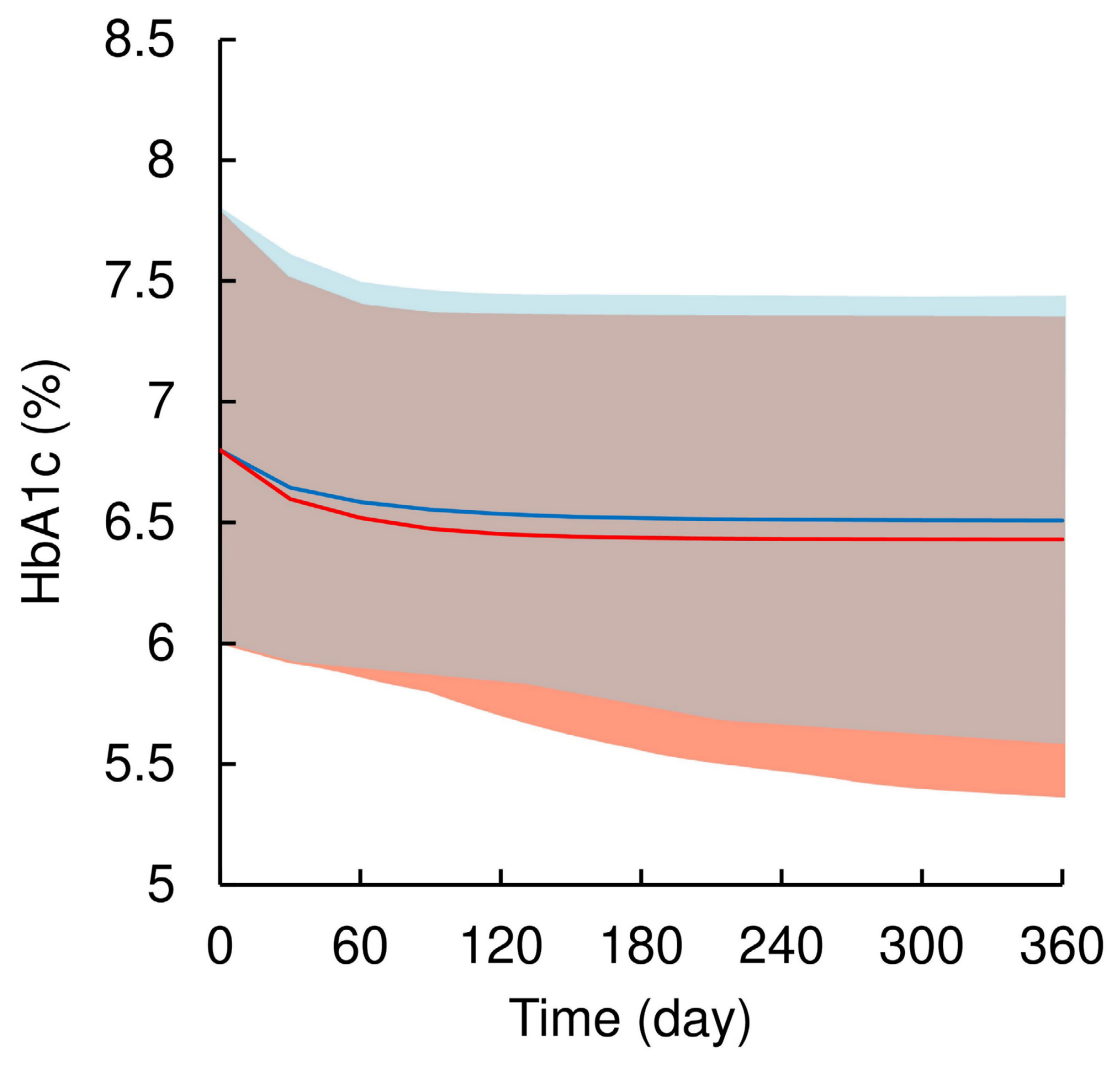
Simulated HbA1c levels after oral administration of 5 or 10 mg/day dapagliflozin during the 12 months of treatment. The solid blue and red line represent the median simulated HbA1c levels after oral administration of 5 or 10 mg/day dapagliflozin, respectively, and the semitransparent red field represents a simulation-based 95% confidence interval for the median. The observed and simulated 5% and 95% percentiles are presented with dashed black and blue lines, respectively, and the 95% confidence intervals for the corresponding model predicted percentiles are shown as semitransparent blue fields.

Figure 4 illustrates the simulated time profiles of HbA1c levels after treatment with 5 or 10 mg of dapagliflozin for 1-year. Dapagliflozin treatment decreased baseline HbA1c levels by 6.8% (5 to 95% range: 6.0 to 7.8%) to 6.5% (5.6%-7.4%) and 6.4% (5.4%-7.4%), respectively. The simulations confirmed that 10 mg dapagliflozin treatment had an almost maximal HbA1c-lowering effect.

## Discussion

This study provides new insights through a population PK-PD model analysis of dapagliflozin using long-term clinical data in a real-world setting. To the best of our knowledge, this study is the first to perform a population PK-PD model analysis of dapagliflozin using real-world data. This model analysis facilitated a comprehensive understanding of the complex relationship between the exposure and response to dapagliflozin. In our previous study, patients with higher HbA1c levels at baseline had greater reductions in HbA1c levels after dapagliflozin treatment than those with lower HbA1c levels 5. The population PK-PD model used in this study successfully characterized this highly significant effect of HbA1c levels at baseline on hypoglycemic effects. Notably, the identification of body weight as a factor influencing the CL/F of dapagliflozin and the finding of large inter-individual differences in t_1/2_HbA1c are outstanding contributions to the field. The strength of this study lies in its pharmacometric approach using long-term real-world data to examine the HbA1c-lowering effects of dapagliflozin.

There was a large inter-individual variability in the time profiles of plasma dapagliflozin concentrations after a once-daily oral dose of 5 mg dapagliflozin (Figure 1). A one-compartment model with first-order absorption could well describe the trough levels of dapagliflozin using previously reported parameters of ka and V/F 11. Interestingly, the current covariate analysis revealed that the sequential change in body weight during dapagliflozin treatment had a significant impact on the CL/F of dapagliflozin, supporting findings from prior clinical trial-based PK models 13. Considering with the small sequential change of body weight as median -1.5 kg (range: -10.8 to 6.0 kg) in the current real-world data, the impact of body weight on systemic exposures of dapagliflozin would be clinically negligible. Unlike previous study 13, neither renal function nor sex significantly affected CL/F, which may be attributed to the limited sample size and single-center study design. Although the model diagnostics using pc-VPC and bootstrap procedure indicated a good description of the observed data, further external validation with a larger and more diverse dataset is warranted to improve model robustness and identify additional covariates affecting drug exposure.

The population PK-PD model for describing HbA1c-lowering effect of a SGLT2 inhibitor has been developed using clinical trial data of canagliflozin 8. In the present study, this model was adapted to analyze the time-profiles of HbA1c levels after the treatment with dapagliflozin. This population PK-PD model is proven to quantitatively describe the HbA1c-lowering effect, and the values of PD parameters can be used to enhance the understanding of the onset and/or degree of the effects. The estimated value of t_1/2_HbA1c (16.1 day) (Table 3) was shorter than that in a previous report (28.2 day) 8. Although the half-life of HbA1c is generally influenced by the erythrocyte lifespan (approximately 120 days) 17, the discrepancy between our estimate and the previous report may be attributed to several factors, including individual variability in HbA1c kinetics, co-administration drugs, and other physiological processes. Additionally, the current population PK-PD model analysis revealed a large inter-individual variability in the t_1/2_HbA1c value. According to the kinetics of HbA1c and plasma glucose level, the half-life of HbA1c is influenced by glycemic improvement time and glycemic control 17. The t_1/2_HbA1c is faster when blood glucose levels improve rapidly with drug administration and when blood glucose control is maintained with good medication adherence. Therefore, the results of the present population PK-PD model analysis indicate that there is large interpatient variability in the glycemic improvement effect time and/or glycemic control. These results suggest that early improvement of blood glucose, good medication adherence, and maintenance of glycemic control are important to reduce inter-individual differences in hypoglycemic effects and to achieve superior HbA1c-lowering effects from the early stage of dapagliflozin treatment. The time to glycemic improvement and glycemic control are candidate covariates in the population PK-PD analysis, and future studies incorporating these candidates into models may elucidate large individual differences in drug efficacy in real-world settings.

Based on the simulation from the population PK-PD model, the predicted HbA1c-lowering effect of 10 mg once-daily dapagliflozin was slightly greater than or comparable to that of 5 mg. In Japan, dapagliflozin is administered at 5 mg once daily to T2DM patients, but information on its hypoglycemic effect when increased to 10 mg once daily is insufficient. Although the current simulation is roughly predicted from the data of patients treated with 5 mg dapagliflozin, the HbA1c level was simulated to decrease to 6.4% (5.4%-7.4%) in the 10 mg dapagliflozin treatment, indicating an almost maximum HbA1c-lowering effect. These findings suggest that the benefits of 10 mg dapagliflozin for chronic heart failure or chronic kidney disease may not be attributable solely to its hypoglycemic action. Further studies on the efficacy and safety of 10 mg dapagliflozin in Japanese patients are required in real-world settings.

The current study had some limitations. First, PK and PD data were obtained at restricted doses during medical procedures. Second, the PK model relied on trough concentrations, reducing the ability to fully characterize absorption kinetics. However, such limitations exist in the analysis of real-world data. Despite model validation efforts, external validation using independent datasets is necessary to confirm our findings. Third, the impact of potential drug-drug interactions on the PK and PD of dapagliflozin was not examined. Fourth, the simulation of HbA1c-lowering effects did not account for potential differences in patient characteristics between the 5 mg and 10 mg dapagliflozin groups. In clinical practice, dose escalation is typically considered based on the patient's response and background, such as body weight or the degree of glycemic control. Therefore, simulations assuming uniform patient backgrounds may not fully reflect real-world variability in treatment responses. Finally, the current study was not a multicenter trial, and the clinical data were obtained from a single-center, single-arm study, which could potentially bias the data. Despite these limitations, this study represents an important step toward understanding the real-world PK and PD of dapagliflozin. Future studies should explore additional covariates affecting treatment response and refine population PK-PD models to facilitate precision medicine approaches in T2DM management.

## Conclusions

This study identified key factors influencing the HbA1c-lowering effects of dapagliflozin and inter-individual variability in glycemic response using a population PK-PD modeling approach with long-term real-world data. Body weight was identified as a significant covariate influencing CL/F, although its clinical impact on drug exposure appears minimal. Substantial inter-individual variability was observed in HbA1c response, suggesting that factors beyond dapagliflozin exposure, such as glycemic improvement rate and adherence, play a critical role in treatment outcomes. While the model-based simulations predicted a relatively greater HbA1c reduction with 10 mg dapagliflozin than with 5 mg, further studies are needed to confirm the clinical relevance of dose escalation in Japanese T2DM patients. This study underscores the utility of pharmacometric modeling with real-world data in optimizing individualized diabetes treatment strategies.

## Supplementary Material

Supplementary figures and tables.

## Figures and Tables

**Table 1:** Baseline patient characteristics

Characteristics	No.	Mean ± SD	Median	Range (min-max)
Patients (persons)	85			
Male-to-female ratio (male/female)	61/24			
Age (years)		59.1 ± 10.0	59	37 - 75
Duration of incidence (years)		11.2 ± 7.8	10.5	1.0 - 33.0
Height (cm)		165.9 ± 8.1	167.0	148.0 - 186.0
Body weight (kg)		78.1 ± 13.4	77.0	49.0 - 118.0
BMI (kg/m²)		28.3 ± 3.5	27.7	22.3 - 38.3
HbA1c (%)		6.8 ± 0.5	6.8	5.6 - 8.8
Systolic Blood Pressure (mmHg)		129 ± 16	129	97 - 166
Diastolic blood pressure (mmHg)		74 ± 12	76	50 - 101
eGFR (mL/min/1.73 m²)		74.6 ± 18.5	74.3	39.2 - 137.8
sCr (mg/dL)		0.81 ± 0.23	0.80	0.32 - 1.50
AST (IU/L)		29 ± 20	22	12 - 140
ALT(IU/L)		36 ± 31	24	10 - 200
Diabetic nephropathy (persons)	28			
Hypertension (persons)	59			
Hypercholesterolemia (persons)	67			

BMI, body mass index; HbA1c, hemoglobin A1c; eGFR, estimated glomerular filtration rate; sCr, serum creatinine; AST, aspartate aminotransferase; ALT, alanine aminotransferase.

**Table 2:** Parameter estimates of the population pharmacokinetic model

Parameters	Final model			Bootstrap (n = 1000)	
	Estimate	CV%		Median	2.5^th^-97.5^th^ percentiles
Population mean (θ)					
k_a_ (1/day)	57.4	Fix		57.4	Fix
CL/F = θ_CL/F_* (BW/77.0)^θ_BW_					
θ_CL/F_ (L/day)	229.3	1.5		229.6	222.6 - 236.4
θ_BW_	0.41	21.3		0.40	0.22 - 0.58
V/F (L)	73.9	Fix		73.9	Fix
Inter-individual variability (ω)					
ω_CL/F_ (%)	13.9	21.3		13.7	11.2 - 16.2
Residual variability (σ)					
σ (%)	39.8	5.4		39.8	35.6 - 44.1

k_a_, first-order absorption rate constant; CL/F, apparent clearance from plasma compartment; V/F, apparent volume of distribution in plasma compartment; BW, body weight.

**Table 3:** Parameter estimates of the population pharmacokinetic-pharmacodynamic model

Parameters	Final model			Bootstrap (n = 1000)	
	Estimate	CV%		Median	2.5^th^-97.5^th^ percentiles
Population mean (θ)					
t_1/2_HbA1c (day)	16.1	4.1		16.0	15.3 - 16.5
E_max_ (HbA1c %/day)	0.034	3.1		0.034	0.031 - 0.035
EC_50_ (ng/mL)	23.7	5.8		21.9	13.5 - 24.4
Inter-individual variability (ω)					
ω_t1/2 HbA1c_ (%)	103.9	11.2		104.1	101.7 - 106.4
Residual variability (σ)					
σ (HbA1c %)	0.24	5.2		0.24	0.21 - 0.27

t_1/2_HbA1c, half-life of HbA1c; E_max_, maximum HbA1c-lowering effect of dapagliflozin for a typical patient with HbA1c at baseline of 8.0%; EC_50_, dapagliflozin plasma exposure at which half-maximal effect is reached.

## Abbreviations

AIC: Akaike's information criterion
ALT: alanine transaminase
AST: aspartate aminotransferase
CL/F: apparent clearance
CV: coefficient of variation
eGFR: estimated glomerular filtration rate
HbA1c: glycated hemoglobin
ka: absorption rate constant
LC-MS/MS: liquid chromatography-tandem mass spectrometry
PD: pharmacodynamic
PK: pharmacokinetic
pc-VPC: prediction-corrected visual predictive check
SGLT2: sodium-glucose cotransporter 2
SD: standard deviation
t_1/2_HbA1c: half-life of HbA1c
T2DM: type 2 diabetes mellitus
V/F: apparent volume of distribution
-2LL: -2 × log likelihood
γ-GTP: γ-glutamyltransferase

## References

[1] Wiviott SD, Raz I, Bonaca MP (2019). Dapagliflozin and Cardiovascular Outcomes in Type 2 Diabetes. N Engl J Med.

[2] McMurray JJV, Solomon SD, Inzucchi SE (2019). Dapagliflozin in Patients with Heart Failure and Reduced Ejection Fraction. N Engl J Med.

[3] Heerspink HJL, Stefánsson BV, Correa-Rotter R (2020). Dapagliflozin in Patients with Chronic Kidney Disease. N Engl J Med.

[4] van der Hoek S, Koomen JV, van Bommel EJM (2023). Exposure-Response Analysis of the Sodium-Glucose Cotransporter-2 Inhibitors Dapagliflozin and Empagliflozin on Kidney Hemodynamics in Patients with Type 2 Diabetes. J Pers Med.

[5] Hayakawa T, Kato KI, Kobuchi S, Kataoka K, Sakaeda T (2022). Associations of Plasma Concentration Profiles of Dapagliflozin, a Selective Inhibitor of Sodium-Glucose Co-Transporter Type 2, with Its Effects in Japanese Patients with Type 2 Diabetes Mellitus. Pharmaceuticals (Basel).

[6] Darwich AS, Ogungbenro K, Vinks AA (2017). Why has model-informed precision dosing not yet become common clinical reality? lessons from the past and a roadmap for the future. Clin Pharmacol Ther.

[7] Riggs MM, Seman LJ, Staab A (2014). Exposure-response modelling for empagliflozin, a sodium glucose cotransporter 2 (SGLT2) inhibitor, in patients with type 2 diabetes. Br J Clin Pharmacol.

[8] de Winter W, Dunne A, de Trixhe XW (2017). Dynamic population pharmacokinetic-pharmacodynamic modelling and simulation supports similar efficacy in glycosylated haemoglobin response with once or twice-daily dosing of canagliflozin. Br J Clin Pharmacol.

[9] Mondick J, Riggs M, Kaspers S (2018). Population Pharmacokinetic-Pharmacodynamic Analysis to Characterize the Effect of Empagliflozin on Renal Glucose Threshold in Patients With Type 1 Diabetes Mellitus. J Clin Pharmacol.

[10] Yao X, Zhou J, Song L (2023). A model-based meta analysis study of sodium glucose co-transporter-2 inhibitors. CPT Pharmacometrics Syst Pharmacol.

[11] Melin J, Parkinson J, Hamrén B (2024). Dapagliflozin pharmacokinetics is similar between patients with heart failure with reduced ejection fraction and patients with type 2 diabetes mellitus. Br J Clin Pharmacol.

[12] Kasichayanula S, Chang M, Hasegawa M (2011). Pharmacokinetics and pharmacodynamics of dapagliflozin, a novel selective inhibitor of sodium-glucose co-transporter type 2, in Japanese subjects without and with type 2 diabetes mellitus. Diabetes Obes Metab.

[13] Melin J, Tang W, Rekić D (2022). Dapagliflozin Pharmacokinetics Is Similar in Adults With Type 1 and Type 2 Diabetes Mellitus. J Clin Pharmacol.

[14] van der Aart-van der Beek AB, Koomen JV, Dekkers CCJ (2021). Evaluation of the pharmacokinetics and exposure-response relationship of dapagliflozin in patients without diabetes and with chronic kidney disease. Clin Pharmacokinet.

[15] Matsuo S, Imai E, Horio M, et al.; Collaborators developing the Japanese equation for estimated GFR (2009). Revised equations for estimated GFR from serum creatinine in Japan. Am J Kidney Dis.

[16] de Winter W, DeJongh J, Post T (2006). A mechanism-based disease progression model for comparison of long-term effects of pioglitazone, metformin and gliclazide on disease processes underlying Type 2 Diabetes Mellitus. J Pharmacokinet Pharmacodyn.

[17] Tahara Y, Shima K (1995). Kinetics of HbA1c, glycated albumin, and fructosamine and analysis of their weight functions against preceding plasma glucose level. Diabetes Care.

