# Supplementary material for: Population Pharmacokinetic-pharmacodynamic Model Analysis of Dapagliflozin for HbA1c-lowering Effects in Japanese Patients with Type 2 Diabetes Mellitus using Long-term Real-world Data
Source: Int J Med Sci. 2025 Apr 22;22(10):2333–41. doi: 10.7150/ijms.111519 (PMC12080569; doi:10.7150/ijms.111519)
Supplement: Supplementary file 1 — Supplementary figures and tables. [file ijmsv22p2333s1.pdf]

Supporting Information

International Journal of Medical Sciences

Article title: Population Pharmacokinetic-pharmacodynamic Model Analysis of Dapagliflozin for HbA1c-lowering Effects in Japanese Patients with Type 2 Diabetes Mellitus using Long-term Real-world Data

Authors: Shinji Kobuchi, Shuhei Sakai, Ryosuke Terada, Ken-Ichiro Kato, Tetsuo Hayakawa, and Toshiyuki Sakaeda

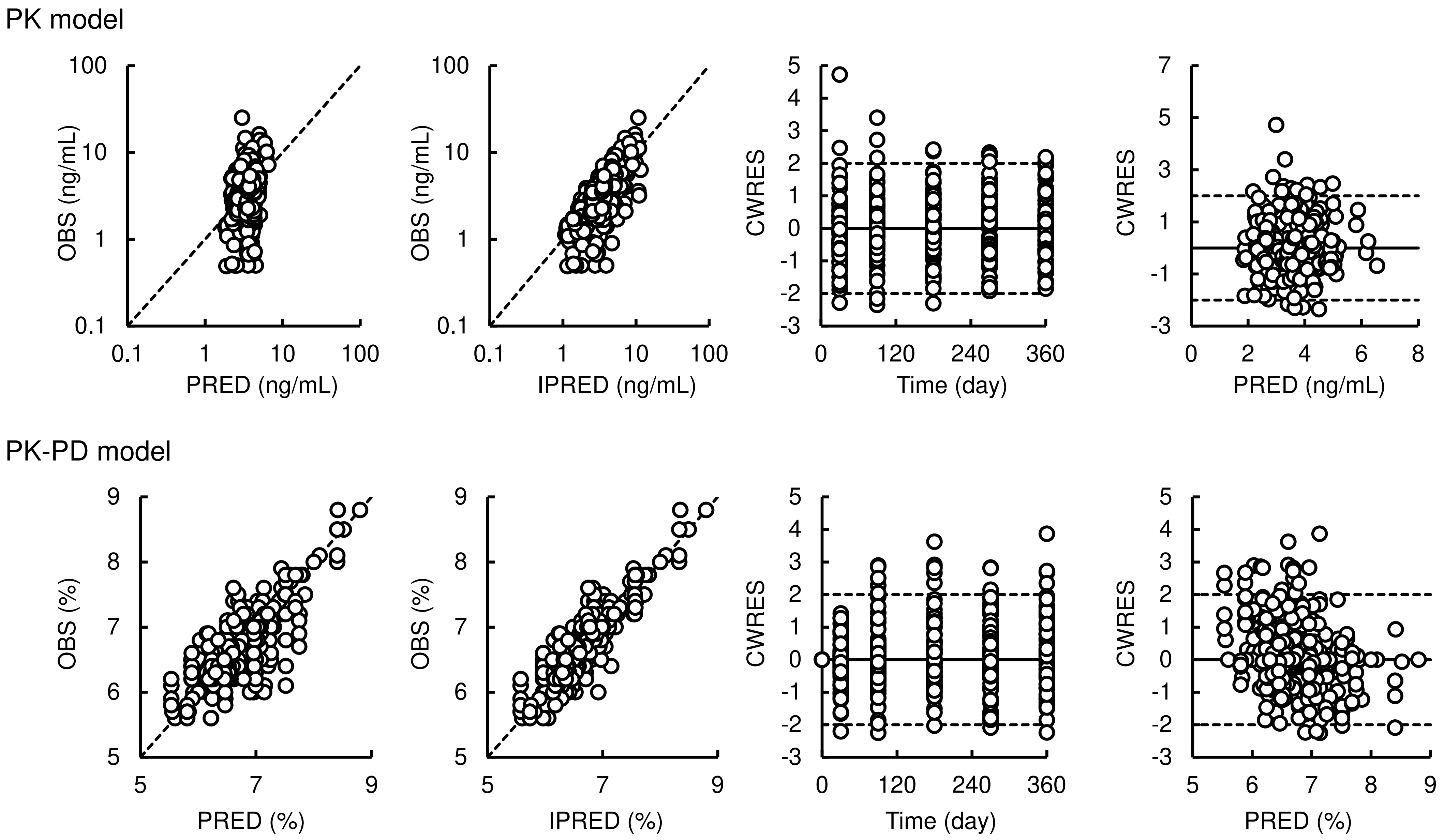

Supplementary Figure S1 Goodness-of-fit plots of final PK and PK-PD model of dapagliflozin for HbA1c. Population predictions (PRED) vs observations (OBS), individual predictions (IPRED) vs OBS, conditional weighed residuals (CWRES) vs time, and CWRES vs IPRED.

Supplementary Table S1 Covariate selection process for the population pharmacokinetics of dapagliflozin

	Model number	Model	No. of parameters	-2LL	Δ-2LL	AIC
Basic model	#0	No covariate	3	1594.9	–	1600.9
Forward addition First step	#1	#1 + CL/F on Age	4	1589.1	−5.8	1597.1
	#2 (Final model)	#1 + CL/F on body weight	4	1576.5	−18.4 ***	1584.5
	#3	#1 + CL/F on height	4	1577.5	−17.4 ***	1585.5
	#4	#1 + CL/F on AST	4	1590.5	−4.4	1598.5
	#5	#1 + CL/F on ALT	4	1590.0	−4.9	1598.0
	#6	#1 + CL/F on eGFR	4	1591.2	−3.7	1599.2
	#7	#1 + CL/F on sex	4	1588.1	−6.8	1596.1
Second step	#8	#2 + Age	5	1576.4	−0.1	1586.4
	#9	#2 + height	5	1573.2	−3.3	1583.2
	#10	#2 + AST	5	1575.0	−1.4	1585.0
	#11	#2 + ALT	5	1575.8	−0.7	1585.8
	#12	#2 + eGFR	5	1575.3	−1.2	1585.3
	#13	#2 + sex	5	1576.0	−0.5	1586.0
Backward elimination	#0	No covariate	3	1594.9	18.4***	1600.9

***$p < 0.001$
